# Metastatic gastric adenocarcinoma to the cutaneous neck and chest wall

**DOI:** 10.1177/2050313X241231515

**Published:** 2024-02-23

**Authors:** Christina M Murphy, Kevin Wang, Christopher Wachuku, Aman Prasad, Ishita Dhawan, Eric E Morgan, Katherine K Brown, Leo Wang

**Affiliations:** 1 Department of Dermatology, University of Pennsylvania, Philadelphia, PA, USA; 2 Rutgers Robert Wood Johnson Medical School, New Brunswick, NJ, USA; 3 Division of Gastroenterology, Cooper University Hospital, Camden, NJ, USA; 4 Department of Pathology and Laboratory Medicine, Hospital of the University of Pennsylvania, Philadelphia, PA, USA

**Keywords:** dermatology, cancer

## Abstract

This case describes an atypical cutaneous presentation of metastatic gastric carcinoma in a patient initially presenting with dysphagia and a sclerotic red plaque overlying the anterior neck and chest. Skin biopsy revealed metastatic adenocarcinoma from the upper gastrointestinal tract. Esophagogastroduodenoscopy revealed stage IV metastatic gastric adenocarcinoma. Treatment with chemotherapy was initiated.

## Introduction

Cutaneous metastasis as a presenting sign of internal malignancy is unusual, accounting for <1% of all new cancer diagnoses.^1^ In patients with metastatic disease, skin metastases occur in less than 10% of all patients and account for less than 2% of malignant tumors that present on the skin.^2–5^ The frequency of metastasis to the skin depends on tumor type and epidemiologic considerations such as sex, age, and risk factors.^3,4^

Gastric carcinoma (GC) is the fourth-leading cause of cancer deaths worldwide and carries a poor prognosis.^6^ Cutaneous metastases of GC account for <1% of all cutaneous metastases. They typically present as slow-growing, firm nodules, such as the so-called “Sister Mary Joseph nodule” affecting the umbilicus. An array of clinical morphologies of cutaneous metastases in GC have been described, such as cellulitis-like erythematous plaques, carcinoma en cuirasse, carcinoma erysipeloides, and other imitators of inflammatory lesions.^7–11^ Histopathologic examination and immunohistochemistry greatly aid in the diagnosis of cutaneous metastases of GC, which may show a variety of different morphologies.^12^

## Case report

A 48-year-old male with no medical history presented to our emergency department with dysphagia and erythematous skin changes overlying the anterior neck and chest. He reported 4 months of worsening rash and a 40-lb weight loss. Prior therapies included multiple courses of antibiotics and a course of methylprednisolone with no improvement.

**Figure 1.**
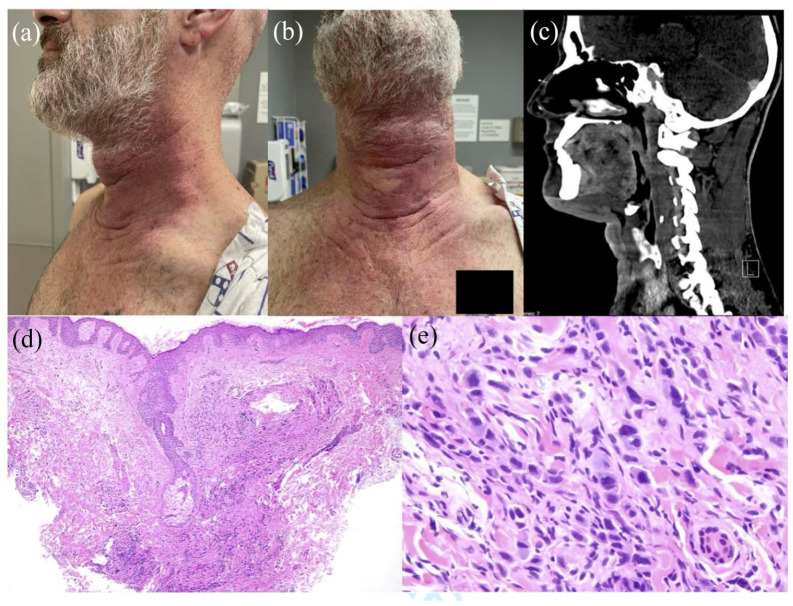
(a and b) Front and side views of erythematous, indurated plaque on neck and anterior chest. (c) Enhancement and thickening of anterior neck on CT imaging. (d) H&E demonstrating diffuse and pleomorphic dermal infiltrate (50x). (e) H&E with pleomorphic dermal infiltrate with cytoplasmic mucin consistent with gastric adenocarcinoma (400×).

On physical examination, an indurated erythematous plaque was noted to overlie the anterior neck extending to the chest. No lymphadenopathy was noted. A Computed tomography scan of the neck was notable for marked skin thickening of the anterior neck and sclerotic foci in several bones. A punch biopsy showed a dermal infiltrate composed of CK7, CDX2-positive tumor cells with cytoplasmic mucicarmine staining, most suggestive of metastatic adenocarcinoma from the upper gastrointestinal tract. Esophagogastroduodenoscopy confirmed the presence of a large submucosal gastric body mass, and the patient was diagnosed with stage IV metastatic gastric adenocarcinoma. Treatment with FOLFOX (leucovorin calcium (folinic acid), fluorouracil, and oxaliplatin) and nivolumab were started (Figure 1).

## Discussion

Diagnosis of cutaneous metastases may be straightforward when a patient with known history of malignancy presents with a rapidly growing lesion. However, in rare cases when cutaneous metastasis is the initial presenting sign of internal malignancy, histopathology becomes critical to accurate and prompt diagnosis. Clinical presentation and site of the lesion can be helpful in diagnosis of cutaneous metastasis and may even lend insight into the type of underlying malignancy. However, these features can be highly variable.

Breast, lung, and colorectal are the most common non-cutaneous malignancies to metastasize to the skin; GC is exceedingly rare and is thought to represent <1% of cutaneous metastases.^3^ GC most commonly metastasizes to the liver, peritoneum, and lymph nodes. Cutaneous metastases are rare, occurring in ~2% of patients with metastatic GC. Cutaneous metastases of GC may appear on the abdomen, face, scalp, chest, back, neck, and extremities.^13–16^ Lesions typically manifest as single or multiple red to violaceous nodules, though they may also present as cellulitis-like or erysipelas-like plaques.^9–11^ On histologic examination, signet ring cells may be an important clue. These cells contain abundant intracytoplasmic mucin that pushes the nucleus to the periphery. However, these are not a specific feature and may be seen in other carcinomas, including breast carcinoma. Immunohistochemical markers may be helpful in making the diagnosis. Specific cytokeratin (CK) expression patterns are useful in determining the origin of metastatic carcinoma from an unknown primary. CDX2, a nuclear transcription factor for intestinal development, is also expressed in gastric and intestinal epithelium as well as adenocarcinoma. Tumor cells with a CK7−/CK20+/CDX2+ immunostaining pattern are typical of colorectal adenocarcinoma. In a study of gastric adenocarcinomas, CK7+/CK20+/CDX2+ was the most common immunostain pattern of expression (21/59, 36%).^17^

## Conclusions

This case represents a striking example of cutaneous metastasis as the presenting sign of GC, which was previously treated for months as a possible infectious or inflammatory issue. The remarkable induration of the skin with accompanying dysphagia is particularly unusual. Skin examination with biopsy was critical to identifying the patient’s underlying malignancy, prompting endoscopy and medical therapy.
